# Wine‐Processed *Polygonatum cyrtonema Hua* Polysaccharides Alleviating Cognitive Dysfunction in CUMS Mice by Regulating Intestinal Microbiota

**DOI:** 10.1002/fsn3.70711

**Published:** 2025-08-08

**Authors:** Jingqiu Zhang, Shuanghui Shi, Junli Zhang, Haiting Zhu, Xiaoyu Wang, Wenjing Chen, Yifan Mao, Mingrui Jiang, Huinan Wang, Xinning Zhang, Haitao Fan, Qiding Zhong, Yingzi Wang

**Affiliations:** ^1^ School of Chinese Materia Medica Beijing University of Chinese Medicine Beijing China; ^2^ College of Bioengineering Beijing Polytechnic University Beijing China; ^3^ Technology Innovation Center of Light Industrial Consumer Goods Quality and Safety for State Market Regulation Beijing China; ^4^ Zhongqing Technology Innovation Center Co. Ltd Beijing China

**Keywords:** cognitive dysfunction, gut microbiota, wine‐processed *Polygonatum cyrtonema Hua* polysaccharide

## Abstract

The incidence rate of cognitive dysfunction (CD) urgently requires the development of safe and effective dietary supplements to prevent and treat this disease. *Polygonatum cyrtonema Hua* (PS) is a traditional Chinese herb that can be used as both food and a medicine, and is known to improve memory and nourish the nerves. However, there has been limited research on the commonly used wine‐processed form of PS (WPS) in clinical practice; consequently, we know little about the pharmacological effects of WPS and the mechanisms involved. In this study, we investigated the homogeneous polysaccharides extracted from PS (SP3) and WPS (JP3) and investigated the effects of JP3 on the CD and the brain–gut axis in chronic unpredictable mild stress (CUMS) mice. SP3 and JP3 showed significant differences in terms of molecular weight, monosaccharide content, and surface morphology. Mechanistic studies showed that JP3 affected the brain–gut axis by regulating the gut microbiota and the intestinal barrier, and improved lesions and oxidative stress in the hippocampus, thus alleviating cognitive impairment. JP3 was able to increase probiotics such as *Lactobacillus murinus* , improve hippocampal lesions, and oxidative stress levels. Compared with inulin, JP3 better promotes the proliferation of *Lactobacillus murinus* and the production of butyric acid and isobutyric acid. The results suggest that WPS homogeneous polysaccharides can regulate intestinal microbiota and improve cognitive level, which is a potential functional product for the prevention of CD.

## Graphical abstract

A homogeneous polysaccharide JP3 with neuroprotective properties was isolated from wine processed *Polygonatum cyrtonema Hua*. JP3 significantly enriched the abundance of *Lactobacillus murinus* in the gut microbiota of CUMS mice. Compared with inulin, JP3 better promotes the proliferation of *Lactobacillus murinus* and the production of butyric acid and isobutyric acid.

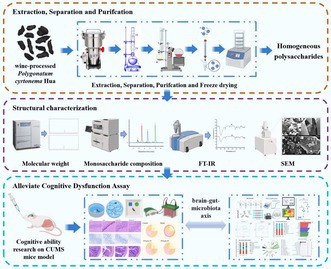

## 1 Introduction

Cognitive dysfunction (CD) refers to the impairment of language, learning, comprehension, and other abilities caused by a variety of factors, and represents a stage between normal aging and dementia (AD) (Cao et al. 2018; Gaugler 2022). The presence of CD indicates that patients have a high risk of developing AD; therefore, their condition holds great significance for the early diagnosis and prevention of AD (DeTure and Dickson 2019; Morris and Cummings 2005). At present, the pathogenesis of CD remains unclear, although researchers have reported the possibility of mutual interaction between gut microbiota and CD, and the “brain–gut–microbe” axis is most likely to be its mediated pathway (Ryu et al. 2019; Dumitrescu et al. 2018). Initially, symbiosis of the gut microbiota leads to the accumulation of reactive oxygen species (ROS) which subsequently leads to the production of activated inflammatory factors as well as neurodegeneration, which is involved in the progression of CD. A balanced gut can indirectly regulate short‐chain fatty acids by directly improving gut microbiota abundance, which will alleviate oxidative stress and other damage (Zorov et al. 2014). More and more discoveries have proved that polysaccharides can effectively regulate the composition of intestinal microbiota and improve brain lesions such as cognitive function by restoring the stability of intestinal microbiota (Silva et al. 2020; Liu et al. 2019). Collectively, the development of polysaccharide‐rich functional food that prevents and improves CD by modulating the gut microbiota is a promising option given the link between CD and the gut microbiota and the beneficial role of polysaccharides in the regulation of gut microbiota.

Traditional Chinese medicine (TCM) is a medicine derived from food and can be used for the early prevention of disease in a safe and effective manner. TCM is considered a promising option for CD (Pei et al. 2020; Liu et al. 2017; Cheung et al. 2015). *Polygonatum cyrtonema Hua* (PS), an edible homologous medicinal material, contains polysaccharides, saponins, flavonoids, and other ingredients, which are known to enhance learning and memory ability, improve mood, delay aging, and are used to treat cognitive impairment (Zhang et al. 2023; Su et al. 2023). The most frequently used method for PS is wine‐processed *Polygonatum cyrtonema Hua* (WPS), which is made by steaming with auxiliary materials such as rice wine. WPS polysaccharides can exert a variety of biological activities, such as antioxidant ability, immune enhancement, and the regulation of intestinal flora (Sun et al. 2020). The molecular weight, monosaccharide composition, glycosidic bond ratio, and advanced conformational characterization, such as the triple helix structure of polysaccharides, are correlated with pharmacological activity and biological function (Yang et al. 2020; Simsek et al. 2023; Mzoughi and Majdoub 2021; Chen et al. 2022, 2023). There are differences in the physicochemical properties and biological functions of PS and WPS polysaccharides, but the structural identification and biological activity of homogeneous polysaccharides in WPS are not sufficiently studied. Furthermore, our previous studies found that WPS was superior to PS in improving immune activity by regulating intestinal microbiota, but we know little about whether WPS polysaccharides play a role in improving the pharmacological activity and mechanism of action of CD by restoring intestinal microbiota balance.

Based on these earlier findings, we will characterize the structure of two homogeneous polysaccharides (SP3 and JP3) purified from PS and WPS. Then, in vitro experiments were used to evaluate the effects of JP3 on antioxidants and the intestinal microbiota. We also investigated the mechanism by which JP3 improves CD through the brain–gut–microbiome axis.

## 2 Materials and Methods

### 2.1 Materials

The stems of *Polygonatum cyrtonema Hua* were obtained from Hunan Province in China. Cellulose diethylaminoethyl (DEAE)‐52 was provided by Shanghai Yuanye Bio‐Technology Co. Ltd. (Shanghai, China). Sephadex G‐100 was obtained from Solarbio Technology Co. Ltd. (Beijing, China). *Lactobacillus murinus* was obtained from BeNa Culture Collection Co. Ltd. (Henan, China). The other chemical reagents used in this paper are analytical grade, and the experimental results are calculated on the basis of dry materials.

### 2.2 Isolation and Purification of SP3 and JP3

Fifty grams of PS and WPS were added to 250 mL of petroleum ether, extracted in a water bath at 80°C for 2 h, the petroleum ether was discarded, and the residue was dried. Add 10 times the amount of deionized water (w/v) to heat and extract three times, 1 h each time. The extract was concentrated to dry at 60°C under reduced pressure, ethanol was added to the final concentration of 80% (v/v). The solution was kept at 4°C for 16 h and deproteinized by the Sevage method. After the solution was concentrated and then freeze‐dried to yield the polysaccharide SP and JP.

The SP and JP polysaccharides on the DEAE‐52 column were eluted sequentially with 0, 0.1, and 0.5 M NaCl. The elution components were collected, their absorbance at 490 nm was measured by the phenol‐sulfuric acid method, and elution maps were prepared. All polysaccharides are labeled as SP1, SP2, SP3, JP1, JP2, and JP3 in elution order. SP3 and JP3 were further purified using Sephadex G‐100 containing 0.5 M NaCl.

The yields of SP3 and JP3 were calculated based on the initial dry weight of the starting materials.

### 2.3 Structural Characterization of SP3 and JP3

#### 2.3.1 Detection of Molecular Weight

The homogeneity of SP3 and JP3 was identified by high performance gel permeation chromatography (HPGPC) under the same conditions. Columns with exclusion sizes of 8 × 10^5^ Da (G5000PWXL; TSK) and 4 × 10^4^ Da (G3000PWXL; TSK) were connected in series. The mobile phase was sodium chloride solution (0.01%) with a flow of 0.6 mL/min.

#### 2.3.2 Monosaccharide Composition Determination

The determination was adjusted based on the methodology of Sun et al. (2020).

#### 2.3.3 Fourier Transform Infrared Spectrum Spectrometer (FT‐IR) Analysis

The transmission FT‐IR spectra of SP3 and JP3 (2 mg in KBr) were collected between 4000 and 400 cm^−1^ using a Fourier transform infrared spectrometer (Thermo Scientific, Carlsbad, USA).

#### 2.3.4 Scanning Electron Microscope (SEM)

A small amount of SP3 and JP3 powder was placed on a conductive adhesive, and after spraying the gold, it was scanned and photographed with SEM (Quanta250; Thermo Fisher Scientific, USA).

#### 2.3.5 In Vitro Simulated Digestion

To evaluate the stability of SP3 and JP3 under simulated gastrointestinal conditions, an in vitro digestion experiment was conducted. The simulated digestion process included both enzymatic and pH adjustments:

- *Gastric digestion*: The polysaccharides were incubated with pepsin at pH 1.5 for 2 h to simulate stomach conditions.
- *Intestinal digestion*: Subsequently, pancreatin and bile salts were added, and the pH was adjusted to 6.8 for another 2 h to simulate intestinal conditions.

Samples were taken at 0, 1, 2, and 3 h to analyze the degradation of the polysaccharides.

### 2.4 Cell Culture and Cell Viability Assay

HT22 cells, purchased from Meilun Biotechnology Corporation (Dalian, China), were cultured in Dulbecco's modified Eagle's medium supplemented with 10% fetal bovine serum, 100 μg/mL streptomycin, and 100 units/mL penicillin at 37°C in a 5% CO_2_ incubator.

HT22 cells were seeded into a 96‐well plate at a density of 1.5 × 10^5^ cells/mL and cultured for 24 h. Cells were pre‐treated with purified polysaccharides SP3 and JP3 at doses of 100, 200, and 400 μg/mL and incubated with 200 mM of H_2_O_2_ for another 24 h. Cell viability was measured as per the methyl thiazolyl tetrazolium (MTT) method.

### 2.5 Congo Red Experiment

One milligram/millilitre of SP3 and JP3 in water were mixed with 80 μg/mL of Congo red, followed by NaOH added to final concentrations of 0, 0.1, 0.2, 0.3, 0.4, and 0.5 mol/L, respectively. The maximum absorption wavelength of the sample mixture was measured using an ultraviolet (UV) spectrophotometer (756PC; SUNNY HENGPING Scientific Instrument Co. Ltd., Shanghai, China) scanning between 400 and 600 nm.

### 2.6 Animals and Experimental Design

Thirty‐two male ICR mice, weighing 20 ± 2 g, were provided by the Sibeifu (Beijing) Biotechnology Co. Ltd. (Beijing, China). The research was conducted according to protocols approved by the institutional ethical committee of Beijing University of Chinese Medicine (approval no.: BUCM‐2023022004‐1195). This study adhered to the ARRIVE guidelines and was conducted in accordance with the U.K. Animals (Scientific Procedures) Act, 1986, and associated guidelines, EU Directive 2010/63/EU for animal experiments, or the National Research Council's Guide for the Care and Use of Laboratory Animals. The conditions for feeding the mice were as follows: ambient temperature 25°C ± 2°C, humidity 60% ± 10%, light and dark cycle for 12 h. Outside of the fasting and water cuts required to build the model, mice had access to feed and water at any time.

After 7 days of acclimatization, mice are randomly divided into four groups of eight each. The four groups are designed as follows: control group (control group); model group (CUMS model group); low‐dose JP3 group (CUMS + 200 mg/kg JP3 group); high‐dose JP3 group (CUMS + 400 mg/kg JP3 group).

#### 2.6.1 Chronic Unpredictable Mild Stress (CUMS) and Treatment

Mice in the stress groups received mild stressors for 6 weeks. The serial repetition of stressors included: wet bedding for 20 h; 45° cage tilting for 24 h; restraint for 2 h; swimming in 4°C water for 5 min; cage shaking for 15 min; nipping tail for 3 min; illuminating for 24 h; removing food or water for 24 h. All these stressors appeared randomly during the experiment and guaranteed the absence of stressors repeated for two consecutive days, and the control group was placed in a normal environment (Xu et al. 2020).

Mice in the low‐dose JP3 group were given 200 mg/kg JP3 and 400 mg/kg JP3 in the high‐dose JP3 group for 4 weeks. Mice in the control group and model group received 0.2 mL of sterile saline by gavage. On the last day of week 6, serum and feces from mice fasted overnight are collected, and intestines and hippocampus are taken after sacrifice.

#### 2.6.2 Behavioral Testing

##### 2.6.2.1 Novel Object Recognition Test (NORT)

The NORT evaluation was conducted in an open‐field apparatus (50 cm × 50 cm × 50 cm). Twenty‐four hours before the start of the formal experiment, all mice undergo a 10‐min training session in the test cassette, exploring the object‐free box and acclimatization. In the first phase of the experiment, each mouse is placed in the middle position facing the wall at the beginning, two identical objects are placed in the corner of the box 10 cm away from each adjacent wall away from the mouse, and the mice are left to explore freely for 10 min before removing the mice. Three hours later, the second phase of the experiment was conducted. One of the old objects is replaced by a new object of similar size, likewise allowing the mouse to explore for 10 min. Clean the box with alcohol each time the mouse is removed. The total time and total number of times the mice touched each object were recorded, and the discrimination index (DI) and recognition index (RI) were calculated. Higher indices represent better learning and memory.

$$\mathrm{DI}=\left(\text{Times}\;\mathrm{new}\;\text{objects were explored}-\text{Times}\;\mathrm{old}\;\text{objects were explored}\right)/\left(\text{Times}\;\mathrm{new}\;\text{objects were explored}+\text{Times}\;\mathrm{old}\;\text{objects were explored}\right)$$

$$\mathrm{RI}=\text{Explore time for}\;\mathrm{new}\;\text{object}/\left(\text{Explore time for}\;\mathrm{old}\;\text{object}+\text{Explore time for}\;\mathrm{new}\;\text{object}\right)$$

##### 2.6.2.2 Morris Water‐Maze Task (MWM)

The water maze was a circular dark pool (60 cm diameter, and 25 cm deep), filled with deionized water (25°C ± 1°C) until the water surface is 15 cm high. The dark pool was divided into four quadrants, and the platform (4 cm width × 12 cm length × 14 cm height) was submerged in the middle of one quadrant. During the 5‐day platform trial testing phase, mice are trained to learn to look for hidden platforms from the middle of the quadrant edge away from the platform, and mice that fail to find the platform within 90 s are guided to stay on the platform. During the procedure, record the time and route of the mouse to the platform. A spatial memory ability test was performed 24 h after the fifth training session. Remove the platform from the pool, depart from the fixed point, and let the mice swim for 60 s. The proportion of time spent in the quadrant where the original platform was located as a proportion of the total swimming time was used as an indicator of spatial memory capacity.

The experimental protocols were performed in accordance with the ARRIVE guidelines, and all animal treatments and experiments were strictly performed with the National Research Council's Guide for the Care and Use of Laboratory Animals and approved by the Beijing University of Chinese Medicine (No. 2020‐SCUEC‐006; Beijing, China).

#### 2.6.3 Hematoxylin and Eosin (H&E) and Nissl Staining

The brain and intestines of mice are placed in 4% paraformaldehyde, stored at 48°C for 48 h, embedded with paraffin, and then cut into glass slides with a thickness of 5 μm and stained with H&E and 1% toluidine blue solution, respectively. Use a microscope to observe these slices. The morphology of Nissl bodies was observed by microscope (Olympus, Tokyo, Japan) and scanned with the NanoZoomer digital scanner (Hamamatsu Photonics K.K., Hamamatsu, Japan).

### 2.7 Oxidative Stress Damage

Oxidative stress damage was evaluated using ROS, superoxide dismutase (SOD), and catalase (CAT) kits (Nanjing Jiancheng Biological Engineering Research Institute, Nanjing, China) according to their instructions, respectively.

### 2.8 Western Blot (WB)

A Western blot was conducted using an established protocol described by (Meng et al. 2023) P Briefly, tissue protein extracts were homogenized in ice‐cold RIPA lysis buffer (Biorigin Inc., Beijing, China) containing protease inhibitor and phosphatase inhibitor (Biorigin Inc., Beijing, China), then separated by 12% SDS‐PAGE and transferred to PVDF membranes. The membrane was then incubated with antibody against Occludin‐1 (Abcam, USA; 1:1000) and claudin‐1 (CLDN1) (Abcam, USA; 1:1000). The obtained signals were normalized to β‐actin (Bioss, Beijing, China; 1:1000). For quantitation of the gray scale by western blot, it was analyzed using Image J software (National Institutes of Health, Bethesda, MD).

### 2.9 16S rRNA Analysis

16S rRNA gene sequencing was determined by Majorbio Biotechnology Co. Ltd. (Shanghai, China). RT‐qPCR has performed to quantify *Lactobacillus murinus* in the fecal samples.

### 2.10 In Vitro Effect of JP3 on the Growth of Probiotic *Lactobacillus murinus*

Microorganisms were stored in glycerol solution at −80°C. The strain should be inoculated onto MRS medium and passaged at least twice until use. Inoculate a single colony of *Lactobacillus murinus* into MRS medium containing 1 mg/mL of JP3. MRS medium without dextrose serves as the control group, and 1 mg/mL inulin medium serves as the positive group. All groups were incubated in an anaerobic environment at 37°C for 48 h. During the cultivation process, determine the optical density value (OD_600_) and pH value of the culture medium at the set time point.

### 2.11 Short Chain Fatty Acids (SCFAs) Analysis

SCFAs produced by probiotics are quantitatively identified by gas chromatography (GC). A series of mixed standard solutions containing acetic acid, propionic acid, butyric acid, isobutyric acid, and valeric acid was prepared using 2‐ethylbutyric acid as the internal standard. Mix the sample with an equal volume of internal standard, and the mixture was analyzed on an Agilent 6890 N GC system equipped with a flame ionization detector (FID) and an Agilent DB‐WAX column (30 m × 0.32 mm × 0.25 μm; Agilent). Nitrogen gas at a rate of 30 mL/min is used as the carrier gas, and the ratio of air, nitrogen, and hydrogen is 10:1:1. The initial column temperature is 60°C, which goes on for 1 min. The temperature rises from 10°C/min to 200°C, which goes on for 13 min. The injection volume is 1 μL.

### 2.12 Statistical Analysis

Experimental data are expressed as mean ± SD, and were analyzed by SPSS software (version 11.0; SPSS, Chicago, IL, USA). Multiple group comparisons that follow a normal distribution are conducted using one‐way analysis of variance, while group comparisons are conducted using the LSD test; nonparametric tests are used if normality is not satisfied. Intestinal microbiota data were analyzed using STAMP software. Values of *p* < 0.05 were considered statistically significant.

## 3 Results

### 3.1 Characterization of SP3 and JP3

#### 3.1.1 Extraction and Isolation of SP3 and JP3

**FIGURE 1:**
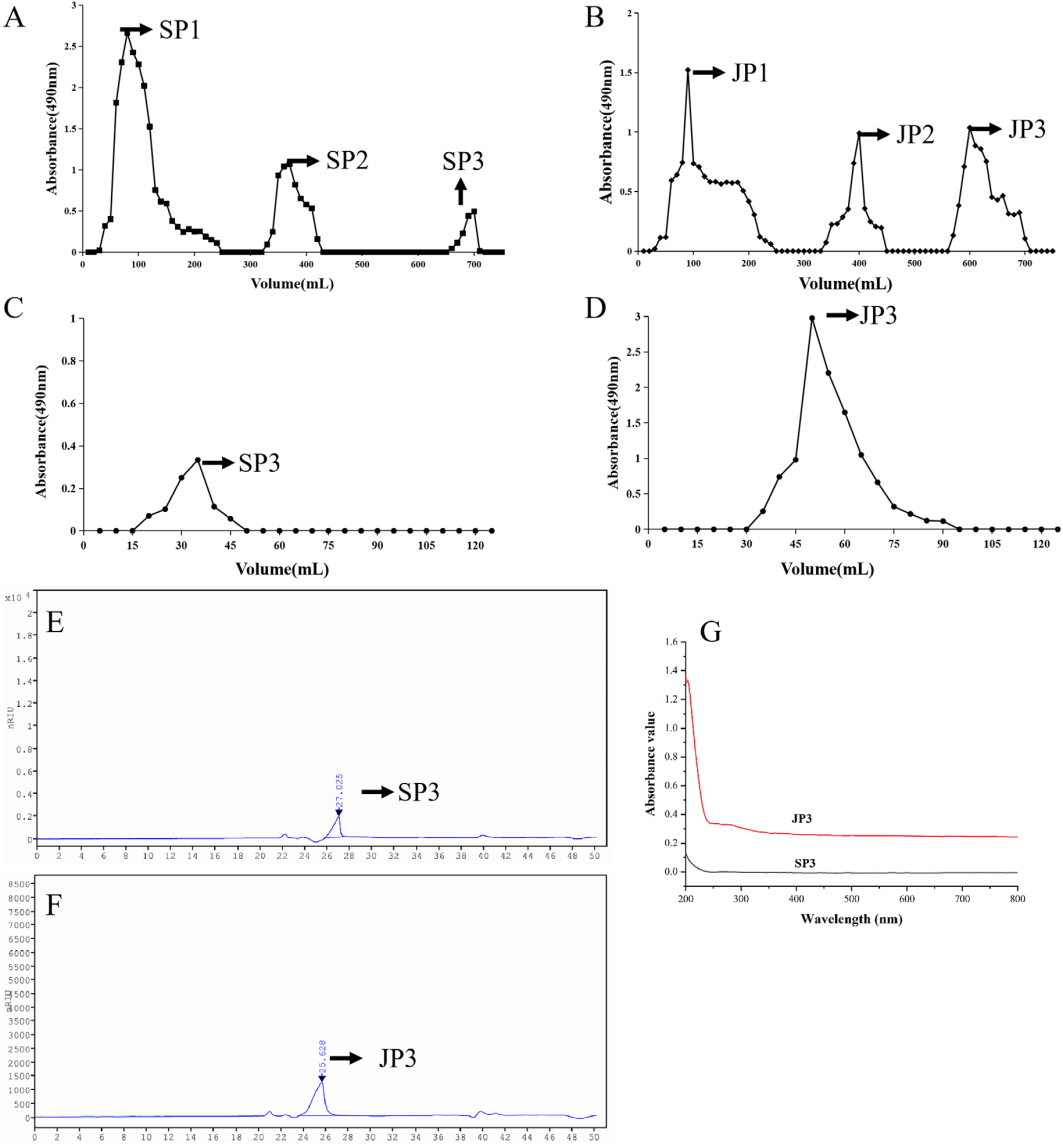
Purification and structural characterization of SP3 and JP3. The separation and elution diagram of PS (A) and WPS (B) on the DEAE‐52 column. SP3 (C) and JP3 (D) were collected from Sephadex G‐100 column. HPLC profile of SP3 (E) and JP3 (F). (G) UV of SP3 and JP3.

The third elution peak of the DEAE‐52 column for SP and WSP was collected (Figure 1A,B), Sephadex G‐100 gel column purification, 0.5 M NaCl elution, and collection of eluates to obtain SP3 and JP3 (Figure 1C,D). The yields are SP3: 12.59% and JP3: 19.12%. The UV scanning spectra of SP3 and JP3 are presented in Figure 1G, which conforms to the characteristics of polysaccharide ultraviolet spectroscopy. No absorbance of SP3 and JP3 was observed around 260 and 280 nm, thus indicating that SP3 and JP3 were of high purity.

#### 3.1.2 Determination of Molecular Weight

HPGPC (Figure 1E,F) showed that the two compounds produced a single symmetrical peak. The Mw/Mn of SP3 was 1.033, while the Mw/Mn of another polysaccharide was 1.114, thus indicating that SP3 and JP3 were homogeneous polysaccharides. Calculated from the standard curve, the average Mw of SP3 and JP3 was calculated to be 34.884 and 83.773 kDa, respectively.

#### 3.1.3 Monosaccharide Composition

SP3 and JP3 were composed of Man, Rha, Glc, Gal, Ara, and GalA in different molar ratios (Table 1). Thus, SP3 and JP3 were considered to be heteropolysaccharides.

**TABLE 1:** Monosaccharide compositions of SP3 and JP3.

Polysaccharide	Peak (t/min)	Monosaccharide	Sugar composition (mol%)
SP3	28.319	Glucose	18.60
	29.758	Galactose	21.81
	24.284	Glucuronic acid	4.74
	25.628	Galacturonic acid	6.58
	23.510	Rhamnose	23.89
	31.019	Arabinose	14.04
	19.429	Mannose	8.20
	17.938	Manuronic acid	0.43
	33.095	Fucose	0.95
	16.931	Guuronic acid	0.76
JP3	28.317	Glucose	10.32
	29.773	Galactose	25.90
	24.314	Glucuronic acid	2.43
	25.631	Galacturonic acid	27.33
	23.533	Rhamnose	22.21
	31.044	Arabinose	8.55
	19.459	Mannose	2.17
	17.938	Manuronic acid	0.11
	33.114	Fucose	0.58
	16.763	Guuronic acid	0.40

#### 3.1.4 FT‐IR Analysis

**FIGURE 2:**
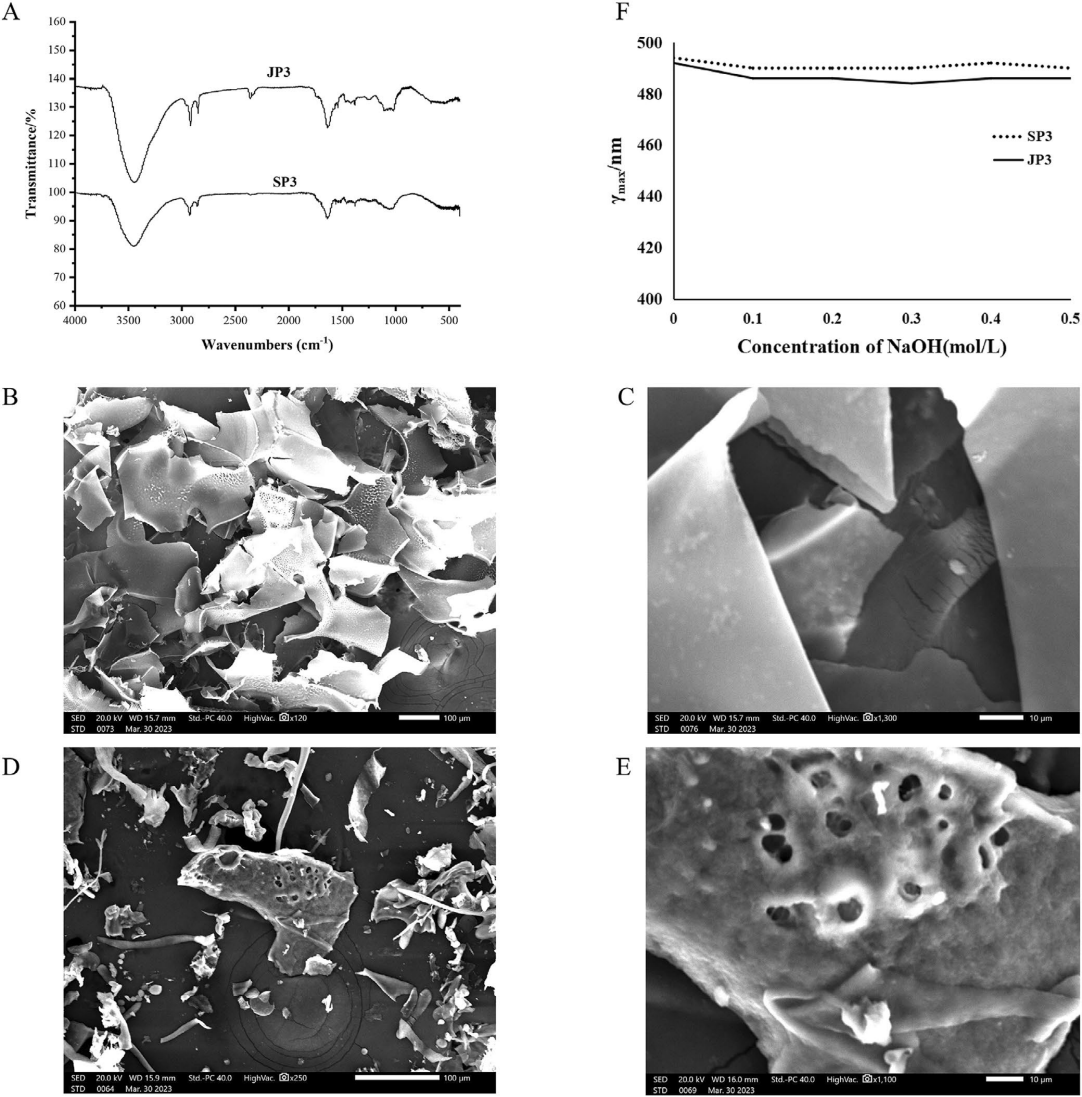
FT‐IR of SP3 and JP3 (A). SEM of SP3: (B) 100 μm, (C) 10 μm. SEM of JP3: (D) 100 μm, (E) 10 μm. The maximum absorption wavelengths of SP3 or JP3 at different concentrations of NaOH (F).

FT‐IR showed a distinct carbohydrate characteristic peak (Figure 2A). The O–H tensile vibration of the hydroxyl group exhibits a distinctly wide absorption peak in the range of 3500–3000 cm^−1^, 1150–1030 cm^−1^ and 1430–1390 cm^−1^ suggest the presence of both JP3 and SP3 α‐ and β‐glycosidic bonds.

#### 3.1.5 SEM

The surface topographies of SP3 and JP3 were investigated by SEM at different scales (100 and 10 μm) (Figure 2B–E). At 100 μm, SP3 exhibited an irregular sheet shape with a relatively smooth surface, varying in shape and size, and with fragmented edges. At 10 μm, irregular cracks were observed on the surface of some sheet‐like structures. At 100 μm, JP3 exhibited irregular sheets, rods, and dots shapes, with uneven surfaces and numerous pores and debris. Partial overlap was observed at 10 μm. These results indicated that the surfaces of polysaccharides varied markedly; this may have contributed to the differences in their biological activities.

#### 3.1.6 Congo Red Analysis

The maximum absorption wavelength (*λ*_max_) of the sample group was similar when the concentration of NaOH exceeded 0.5 mol/L, thus demonstrating that SP3 and JP3 did not have a triple helix structure (Figure 2F).

#### 3.1.7 In Vitro Simulated Digestion Results

The total carbohydrates and reducing sugars of JP3 during the simulated digestion process are shown in Table 2. There was no significant change in the total sugar content and reducing sugar content of JP3 during the simulation of artificial gastric and intestinal fluids (*p* > 0.05).

**TABLE 2:** Simulate changes in total and reducing sugar content during digestion.

	Total sugar content (%)	Reducing sugar content (%)
Gastric		
0 h	12.32 ± 1.48	0.015 ± 0.0041
1 h	12.15 ± 1.36	0.019 ± 0.0019
2 h	11.40 ± 2.18	0.017 ± 0.0041
3 h	11.67 ± 0.88	0.014 ± 0.0091
Intestinal		
0 h	11.24 ± 1.67	0.009 ± 0.0034
1 h	11.79 ± 2.05	0.013 ± 0.0074
2 h	11.35 ± 1.49	0.011 ± 0.0066
3 h	11.42 ± 1.74	0.016 ± 0.0059

### 3.2 Neuroprotective Effect of SP3 and JP3

**FIGURE 3:**
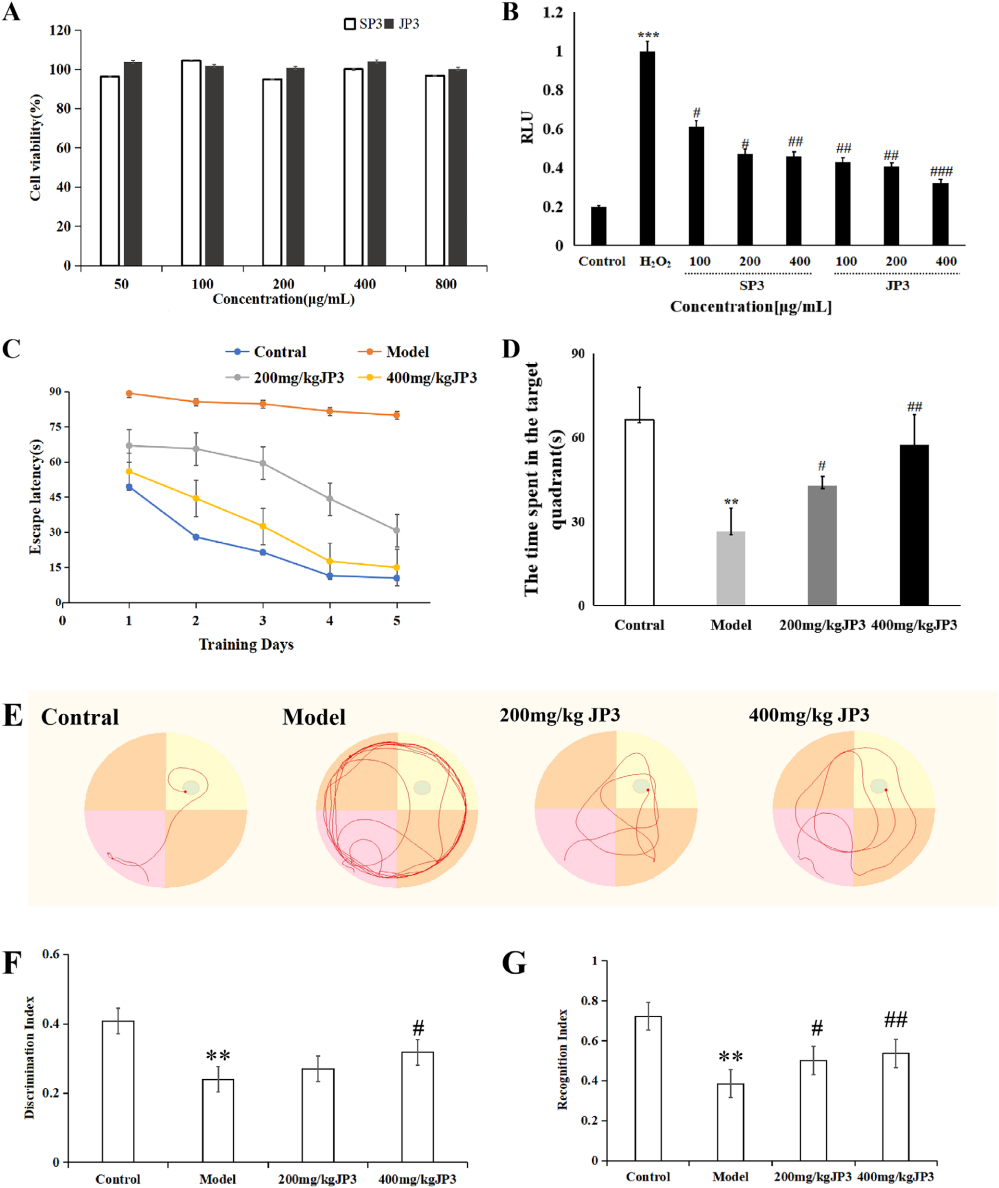
(A) Cell survival rate curves of SP3 and JP3. (B) Effects of SP3 and JP3 on cell levels of ROS. (C) The total escape latency in each training trial day was shown. (D) The time spent in the target quadrant. (E) Mice swimming patterns among different groups. DI (F) and RI (G) of different groups.(** *p* < 0.01, ***p < 0.001, compared with the Control group; ^#^ *p* < 0.05, ^##^ *p* < 0.01, ^###^ *p* < 0.001, compared with the Model group)

SP3 and JP3 had no significant cytotoxicity to HT‐22 cells in the range of 50–800 μg/mL (Figure 3A), and the safety profile was good. Figure 3B shows that the model group significantly increased the production of ROS in H_2_O_2_‐induced HT‐22 cells compared to the normal group (*p* < 0.001). The ROS levels decreased after treatment with SP3 and JP3 at concentrations of 100, 200, and 400 μg/mL, and the formation of ROS was significantly inhibited by SP3 at 400 μg/mL and JP3 at 200 μg/mL (*p* < 0.01). The results showed that JP3 could dependently inhibit the level of ROS in HT22 cells induced by H_2_O_2_, and JP3 had a better protective effect on HT22 cells than SP3. Therefore, JP3 was selected to further study the effect of CD.

### 3.3 JP3 Resisted Cognitive Decline in CUMS Mice

In the MWM test, on day 5, CUMS mice took longer to ascend the platform when compared to mice in the control group (Figure 3C–E). Furthermore, the 200 and 400 mg/kg JP3 groups showed lower escape latency, especially in the 400 mg/kg JP3 group on day 5 (*p* < 0.001). These results showed that different doses of JP3 could improve the long‐term memory of CUMS mice, and that the improvement effect of JP3 in the high‐dose group was more significant.

The NOR test showed that compared with the control group mice, the CUMS mice had a significantly reduced time and number of explorations for new objects (*p* < 0.05), thus indicating that these mice had impaired short‐term memory. The performance of the mice tended to be normal (*p* < 0.01) when administered 400 mg/kg of JP3 (Figure 3F,G).

### 3.4 Histopathology

**FIGURE 4:**
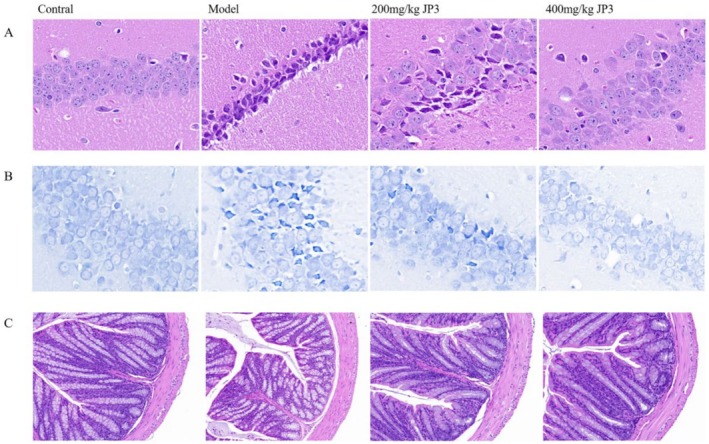
Histopathology of hippocampus (A, B) and gut (C).

H&E staining showed that compared with the control group, the neurons in the hippocampus tissue of the model group were shrunken, the staining was deeper, the cytoplasmic demarcation of the nucleus was unclear, and the cell polarity in the hippocampus had disappeared, separated scattered (Figure 4A). The tissue lesions in the different dose groups were improved after JP3 treatment, and the neuronal morphology in the high‐dose JP3 group was almost regular; the cytoplasmic demarcation of the nucleus was relatively clear, and the overall neuronal condensation in the hippocampus was reduced.

Nissl bodies were detected by Nissl staining for neurons in the CA1 region of the hippocampus. In the CUMS group, hippocampal neurons were disordered, and some neurons were atrophied. In addition, their nits are reduced or absent. Following JP3 treatment, the hippocampus of mice showed obvious Nissl bodies, and the morphology was more similar to that of the control group (Figure 4B).

H&E staining further showed that when compared with the control group, the model group had a significantly thinner colonic muscle layer, inflammatory cell infiltration, local edema, crypt damage, and irregular cell arrangement. Treatment with different doses of JP3 improved the muscularis of the colon, alleviated mucosal injury, and improved the cell arrangement (Figure 4C).

Figure 4A represents H&E staining of hippocampal tissue showing neuronal morphology in different groups. Figure 4B represents Nissl staining of the CA1 region of the hippocampus highlighting the presence of Nissl bodies. Figure 4C represents H&E staining of colon tissue showing histopathological changes in different groups. Compared with the control group, the expression of occludin and CLDN1 in the colon of the model group mice was significantly reduced (*p* < 0.01), indicating impaired intestinal barrier in CUMS mice. Low and high doses of JP3 administration significantly increased the protein expression of occludin and CLDN1 (Figure 4D).

### 3.5 JP3 Reshaped the Gut Microbiota in CUMS Mice

**FIGURE 5:**
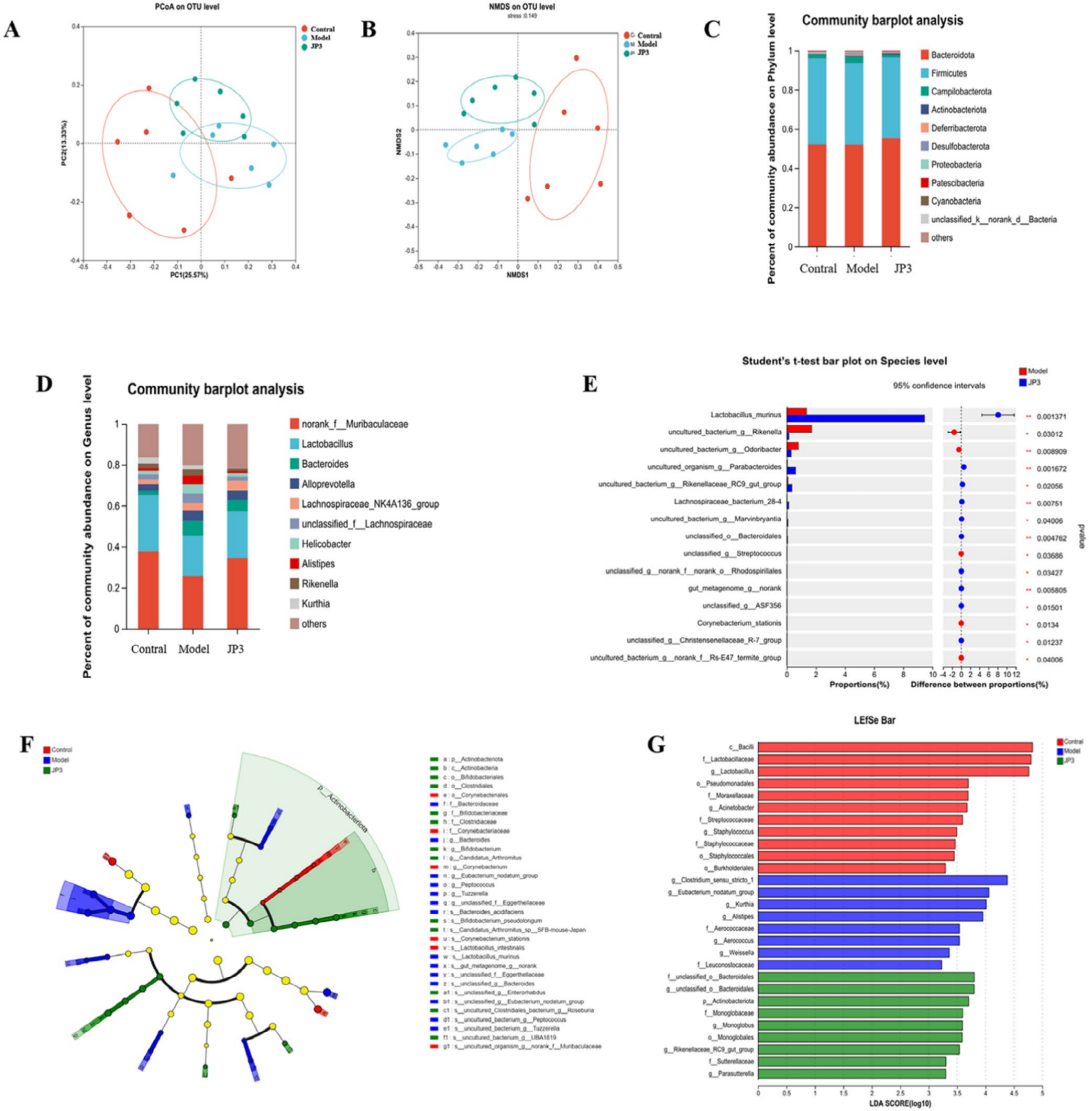
(A) PCoA analysis. (B) NMDS analysis. Relative abundance of gut microbiota at the phylum levels (C) and genus levels (D). (E) The differences of the gut microbiota at the genus levels between model and JP3. LEfSe result included the cladogram (F) and main dominant bacteria (G).

As shown in Table 3, there were significant differences between the Shannon index and Simpson index of gut microbiota abundance in different mice; furthermore, these two indicators were significantly lower in the model group when compared to the control group (*p* < 0.001). Principal coordinate analysis (PCoA) showed that the diversity of intestinal microbiota in the JP3 group was closer to that of the control group than that of the model group; the NMDS results followed the same trend (Figure 5A,B).

**TABLE 3:** Alpha diversity analysis of samples.(*p < 0.05, compared with the Control group; ^#^ *p* < 0.05, ^##^ *p* < 0.01, compared with the Model group).

	Shannon	Simpson	Ace	Chao
Control	4.01 ± 0.25	0.045 ± 0.013	484.37 ± 68.77	481.43 ± 70.06
Model	3.80 ± 0.31	0.058 ± 0.015*	460.60 ± 57.94*	455.14 ± 60.48*
400 mg/kg JP3	4.07 ± 0.28	0.041 ± 0.010^#^	536.94 ± 55.88^#^	532.56 ± 52.41^##^

The composition of the gut microbiota of different groups was then analyzed from the perspectives of phylum and genus. As shown in Figure 5C–F, Firmicutes and Bacteroidota were the main phyla constituting the main types of intestinal microbiota in each group, and the F/B levels in the JP3 group were closer to those in the blank group. At the genus level, JP3 intervention significantly increased the proportion of *Lactobacillus* from 19.65% to 22.79% (*p* < 0.05) and reduced the abundance of *Alistipes* from 7.40% to 5.56% (*p* < 0.05)when compared to the model group. In addition, linear discriminant analysis (LDA) effect size analysis (LEfSe) was performed to reveal specific bacterial populations for JP3 intervention. Compared to the control group, the contents of *Lactobacillus murinus* , *Lactobacillus*, *Alloprevotella*, and *Lachnospiraceae_NK4A136_group* in JP3 were abundant while *Bacteroides*, *Helicobacter*, and *Alistipes* were significantly lower (LDA > 3).

To further quantify the abundance of *Lactobacillus murinus* , we performed RT‐qPCR using specific primers targeting the 16S rRNA gene of *Lactobacillus murinus* . The results showed a significant increase in the abundance of *Lactobacillus murinus* in the JP3‐treated group compared to the model group (*p* < 0.01). This finding supports the role of JP3 in selectively enriching beneficial gut microbiota.

### 3.6 Oxidative Stress Damage

**FIGURE 6:**
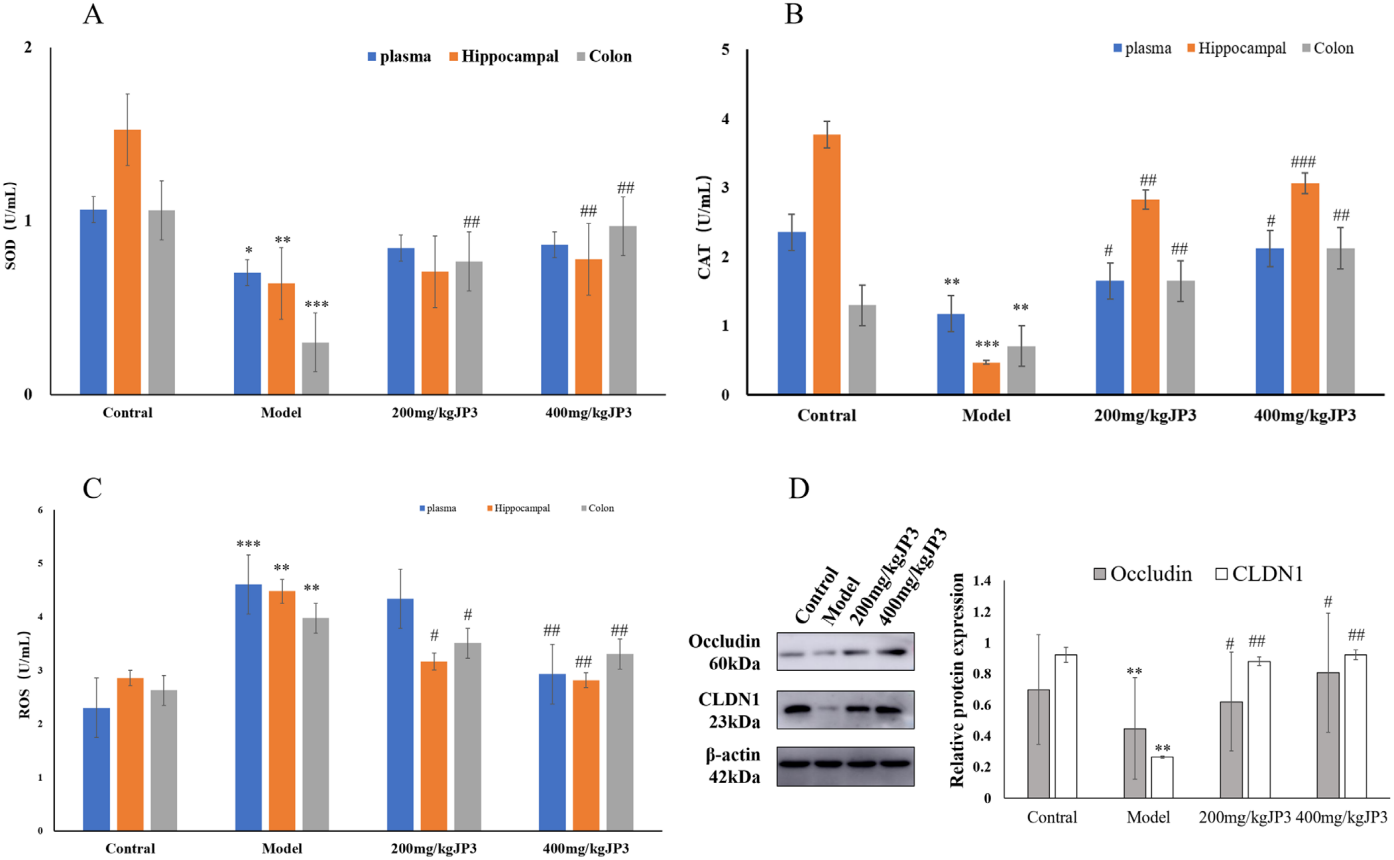
Effects of JP3 on expression of SOD (A), CAT (B), and ROS (C) in hippocampus, plasma, and colon of CUMS mice. The protein expression levels of Occludin and CLDN1 (D). *p < 0.05, **p < 0.01, ***p < 0.001 compared with the control group; ^#^ *p* < 0.05, ^##^ *p* < 0.01, ^###^ *p* < 0.001 compared with the model group.

ROS‐induced oxidative damage can lead to apoptosis and neuroinflammation, which are closely related to the pathological process of neurodegenerative diseases. Compared with the control group, the levels of ROS in the hippocampus, plasma, and intestinal tract of mice in the model group showed an upward trend (*p* < 0.01). This increase was significantly inhibited by JP3 at a dose of 400 mg/kg (*p* < 0.01). Moreover, the levels of antioxidant enzymes, including SOD and CAT, in the model group were significantly reduced (*p* < 0.05). The levels of SOD and CAT in intestinal tissues were significantly increased after treatment with 200 mg/kg of JP3 (*p* < 0.05) while the levels of SOD and CAT in the hippocampus, plasma, and intestinal tissues were significantly restored in the 400 mg/kg JP3 group (*p* < 0.01) (Figure 6A–C).

### 3.7 WB

To further investigate the effect of JP3 on intestinal barrier integrity, the levels of tight junction protein were measured in the control and treatment groups of mice. The results showed that compared with the control group, the levels of Occludin and CLDN1 in the model group were significantly reduced (*p* < 0.01). On the contrary, JP3 significantly increased the levels of Occludin and CLDN1 (*p* < 0.05).

### 3.8 Effect of JP3 on the Growth of Selected Probiotics

**FIGURE 7:**
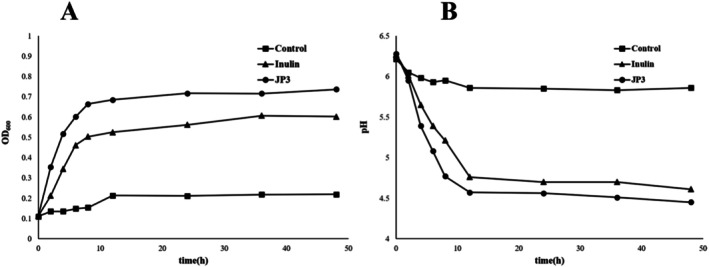
Plot of OD_600_ (A) and pH (B) values with incubation time in media containing different carbon sources of *Lactobacillus murinus* .

The experiment evaluated whether JP3 can enhance the proliferation and acid production ability of selected strains. As presented in Figure 7, the OD_600_ of the inulin and JP3 groups rapidly increased between 2 and 8 h and remained stable after 12 h. Compared with the control group, the OD_600_ of the inulin and JP3 groups was significantly increased (*p* < 0.01); at 48 h, there was a difference in OD_600_ between the JP3 group and the inulin group (*p* < 0.05). The results indicate that JP3 can better promote the proliferation of *Lactobacillus murinus* , and its promoting effect is similar to that of inulin.

### 3.9 Short Chain Fatty Acids

Table 4 illustrates that the total content of SCFAs in the positive group and JP3 group was significantly higher than that in the control group, indicating that *Lactobacillus murinus* can utilize inulin and JP3. Although there was no significant difference in total acid content, the levels of butyric acid and isobutyric acid in the JP3 group were significantly higher than in the positive group (*p* < 0.05).

**TABLE 4:** SCAFs content in different carbon groups ($\bar{x}\pm s$, *n* = 3).

Sample	Concentration (mg L^−1^)					
	Acetic acid	Propionic acid	Isobutyric acid	Butyric acid	*n*‐Valeric acid	Total acid
Blank	35.36 ± 0.14	8.71 ± 0.11	2.61 ± 0.09	6.73 ± 0.21	1.78 ± 0.12	55.19 ± 1.15
Inulin	67.17 ± 0.75^a^	10.62 ± 0.76^a^	2.98 ± 0.20^a^	8.28 ± 0.30^a^	3.56 ± 0.11^a^	92.61 ± 0.15^a^
JP3	67.48 ± 0.59^a^	9.37 ± 0.92^a^	3.76 ± 0.08^a^, ^b^	11.09 ± 1.29^a^, ^b^	3.64 ± 0.04^a^	96.50 ± 2.63^a^

^a^ *p* < 0.05, compared with the blank group.

^b^ *p* < 0.05, compared with the inulin group.

## 4 Discussion

Intestinal flora‐mediated oxidative stress disorders are known to have a negative impact on the development and progression of neurodegenerative diseases (Tse 2017). In previous studies, *PS* extract was shown to exhibit activity against AD lesions and regulate the intestinal flora (Luo et al. 2022). In this study, we purified a homogeneous polysaccharide (SP3 and JP3) with neuroprotective activity from PS and WPS, with a molecular weight of 34.88 kDa for SP3 and 83.77 kDa for JP3. SP3 and JP3 consist of Man, Rha, GlcA, GalA, Glc, Gal, Xyl, Ara, Fuc, GulA, and ManA. Studies have shown that the proportion of uronic acid has an effect on the biological activity of polysaccharides, and there is a positive correlation with antioxidant activity (Li et al. 2021). In this study, JP3, which has a higher proportion of uronic acid, did exhibit better antioxidant activity than SP3. In order to investigate the regulatory effect of the PS homogeneous polysaccharide JP3 on cognitive dysfunction and its potential mechanism of action, we used CUMS‐induced mice with cognitive dysfunction as a research model. The results showed that the CUMS mice showed obvious cognitive dysfunction, and after comparing the behavior, physiology, and biochemistry of mice before and after administration, it was found that JP3 could significantly improve the cognitive ability of CUMS mice in different aspects, alleviate intestinal barrier damage, inhibit oxidative stress levels, and make the composition of intestinal microbiota closer to normal mice. Therefore, we believe that the decrease in the level of oxidative stress caused by the restoration of the balance of the intestinal flora may be involved in this process. We consider that the reduction of oxidative stress levels caused by the restoration of the intestinal flora balance may have participated in this process.

The brain–gut–microbiota axis is considered to be a complex two‐way communication system, which encompasses the role of the gut microbiota in influencing the nervous system through multiple pathways. Previous studies have shown that the gut microbiota plays an important role in the development of cognitive dysfunction by regulating the brain–gut–microbiota axis and oxidative stress levels, so it is considered a possible therapeutic direction for the prevention and treatment of AD (Liu and Zhu 2018; Arneth 2018; Morais et al. 2021). The ratio between Bacteroidetes and Firmicutes is associated with susceptibility to disease states (Liu et al. 2019). Our analyses showed that JP3 supplementation could increase the abundance of Bacteroidetes and decrease the abundance of Firmicutes; the F/B in the JP3 group was better than in the model group. Considering the limitations of experimental conditions and time, this study validated the direct effect of JP3 on gut microbiota by using alternative carbon sources for in vitro cultivation of key bacterial species. These experiments also helped clarify the interactions among the host, microbiota, and metabolites. Therefore, the results of this study on 16S rRNA sequencing data and the improvement of cognitive function and intestinal health in mice provide relevant evidence for the role of JP3 in regulating intestinal microbiota, which will help us plan future FMT and sterile experiments to further validate the above findings.

The differences in gut microbiota at the genus level and LEfSe analysis indicate that JP3 intervention increased the abundance of beneficial microbiota for butyrate production, such as *Lactobacillus*, while inhibiting the abundance of *Bacteroides* that have adverse consequences on CD production. The abundance of *Lactobacillus* has been considered to be negatively correlated with clinical indicators of cognitive function, while *Bacteroides* shows a positive correlation (Guo et al. 2021). At the species level, JP3 specifically unregulated the level of *Lactobacillus murinus* . The in vitro experimental results also showed that the total OD_600_ content of both inulin and JP3 groups was significantly higher than that of the blank group, indicating that both inulin and JP3 can promote the proliferation of *Lactobacillus murinus* , thus suggesting that JP3 improvement of cognitive dysfunction in CUMS mice may be related to the increase in the abundance of *Lactobacillus murinus* . Furthermore, the gut microbiota is accountable for the production of several metabolites, among which SCFAs are of particular concern due to their role in gut health and signaling. Butyric acid and isobutyric acid, as energy sources for colon epithelial cells, can promote the proliferation of probiotics, inhibit the release of inflammatory factors, and improve intestinal barrier function. Although there were no significant differences in total acid content between the inulin and JP3 groups, the JP3 group showed a significant increase in the production of butyric acid and isobutyric acid compared to the inulin group, suggesting that *Lactobacillus murinus* is one of the candidate targets for JP3 treatment of CD, and JP3 can be hydrolyzed by *Lactobacillus murinus* to produce beneficial short‐chain fatty acids for the intestine, thereby improving the intestinal barrier and gut microbiota composition. Studies have shown that PS polysaccharides can improve the relative abundance of Lachnospiraceae in the intestinal microbiota of high‐lipid‐induced cognitive dysfunction in mice, while polysaccharide PSP‐1 derived from *Polygonatum sibiricum* can regulate *Bacteroides acidifaciens* in mice with Alzheimer's disease. These results indicated that JP3, as a homogeneous polysaccharide with definite CD‐relieving effects, has the potential to delay CD via the targeted regulation of *Lactobacillus*, along with inhibiting oxidative stress. *Lactobacillus murinus* synergistically improves hippocampal function through multiple pathways along the gut–brain axis, with core mechanisms involving multiple levels such as SCFA immune regulation and vagus nerve BDNF signaling. In the brain, excessive activation of NF‐κB can lead to neuroinflammation, which in turn damages neuronal function and plasticity in the hippocampus. The enrichment of *Lactobacillus murinus* likely contributes to hippocampal improvements through several mechanisms: SCFA‐mediated NF‐κB inhibition: *Lactobacillus murinus* produces short‐chain fatty acids (SCFAs) such as butyrate, which can inhibit the NF‐κB pathway, reducing inflammation and oxidative stress in the hippocampus. Vagus nerve signaling: SCFAs can also activate the vagus nerve, enhancing brain–gut communication and promoting neuroplasticity in the hippocampus (Li et al. 2018). Enhanced gut barrier function: *Lactobacillus murinus* improves gut barrier integrity, reducing the translocation of inflammatory molecules to the brain (Pan et al. 2024). These mechanisms collectively contribute to the observed cognitive improvements in JP3‐treated mice.

The accumulation of ROS caused by abnormal intestinal flora will increase the levels of oxidative stress markers in the brain such as CAT, SOD, malondialdehyde (MDA) and lipid peroxide (LPO) through the two‐way communication of the brain–gut–gut and microbial axis, and increase the levels of inflammatory factors such as COX‐2, TNF‐α, IL‐1β and other inflammatory factors in the hippocampus, thus further aggravating neurodegeneration, causing damage to the brain cells, metabolism disorders in the nerve cells, degeneration, and even necrosis, thus promoting and participating in β‐amyloid neurotoxicity and aggregation, eventually leading to CD even AD (Dumitrescu et al. 2018; Nago et al. 2021). Prebiotics–gut microbiota interactions are able to regulate the levels of exogenous and endogenous ROS through the production of various metabolites (Wu et al. 2021; Singh et al. 2022). Cognitive impairment in CUMS mice may be related to the neuroinflammation caused by oxidative stress. JP3 has played a positive role in inhibiting ROS levels and restoring antioxidant enzymes in mice. In addition, JP3 can indirectly reduce oxidative stress levels by regulating gut microbiota and affecting the production of short chain fatty acids. This may be one of the potential mechanisms by which JP3 inhibits oxidative damage and alleviates cognitive impairment through brain gut microbiota mediation.

However, further research about the relationship between the specific structure of polysaccharides and their roles in alleviating cognitive dysfunction needs to be explored.

## 5 Conclusion

In this study, homogeneous polysaccharides SP3 (34.884 kDa) and JP3 (83.773 kDa) without triple helix structure were extracted and purified from PS and WPS, respectively. SP3 and JP3 had the same monosaccharide composition, but the proportion of galactose and galacturonic acid is higher. JP3 can alleviate CD in CUMS mice by inhibiting oxidative stress levels, reducing intestinal barrier damage, strengthening mucosal integrity, ameliorating the expression of intestinal epithelial tight junction protein, improving the spatial memory level and cognitive ability. In addition, JP3 restores the diversity of intestinal flora, positively regulates the structural and compositional aspects of the gut microbiota and the production of related metabolites towards antioxidant activity, increases the abundance of beneficial bacteria such as *Lactobacillus* associated with neurotransmitter and butyrate production, and inhibits *Bacteroides* with negative effects. Differential microbial analysis revealed that JP3 selectively increased the abundance of *Lactobacillus murinus* . The results of in vitro culture experiments confirmed that compared to inulin, JP3 gets a better promoting effect on the proliferation and acid production of *Lactobacillus murinus* , effectively increasing the production of butyric acid and isobutyric acid in SCFAs. The results indicate that JP3 has multiple factors in alleviating CD, including inhibiting oxidative stress, reversing histopathological changes, selectively regulating the relative abundance of gut microbiota, and affecting the production of gut microbiota metabolites short chain fatty acids.

## Author Contributions

**Jingqiu Zhang:** conceptualization (equal), formal analysis (equal), investigation (equal), validation (equal), writing – original draft (equal). **Shuanghui Shi:** data curation (equal), writing – original draft (equal). **Junli Zhang:** formal analysis (equal). **Haiting Zhu:** formal analysis (equal). **Xiaoyu Wang:** formal analysis (equal). **Wenjing Chen:** formal analysis (equal). **Yifan Mao:** formal analysis (equal). **Mingrui Jiang:** data curation (equal), formal analysis (equal). **Huinan Wang:** data curation (equal), investigation (equal). **Xinning Zhang:** investigation (equal). **Haitao Fan:** resources (equal), software (equal). **Qiding Zhong:** resources (equal), supervision (equal). **Yingzi Wang:** writing – review and editing, funding acquisition.

## Conflicts of Interest

The authors declare no conflicts of interest.

## Data Availability

The data that support the findings of this study are available from the corresponding author upon reasonable request.
